# 1,2,4,5-Tetra­methyl-3,6-diphenyl-1,2,4,5-tetra­aza-3,6-diphosphinane

**DOI:** 10.1107/S160053680800665X

**Published:** 2008-03-14

**Authors:** Frederik H. Kriel, Judy Caddy, Manuel A. Fernandes

**Affiliations:** a Project AuTEK, Mintek, Private Bag X3015, Randburg 2125, South Africa; b Molecular Science Institute, School of Chemistry, University of the Witwatersrand, PO Wits, 2050 Johannesburg, South Africa

## Abstract

The title compound, C_16_H_22_N_4_P_2_, crystallizes about a centre of symmetry, leading to a chair conformation of the heterocyclic ring as is commonly found for this type of compound.

## Related literature

For related structures, see: Reddy *et al.* (1994 ▶, 1995 ▶).
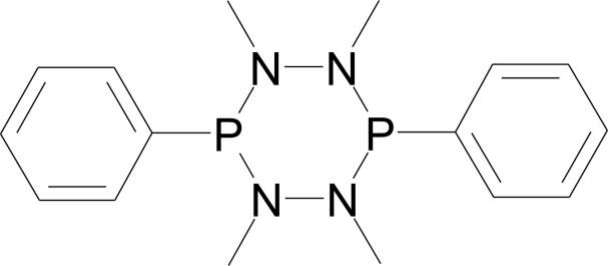


## Experimental

### 

#### Crystal data

C_16_H_22_N_4_P_2_
*M* *_r_* = 332.32
Orthorhombic, 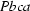

*a* = 13.2879 (16) Å
*b* = 7.5426 (9) Å
*c* = 17.125 (2) Å
*V* = 1716.4 (4) Å^3^
*Z* = 4
Mo *K*α radiation
μ = 0.26 mm^−1^
*T* = 173 (2) K
0.38 × 0.27 × 0.26 mm

#### Data collection

Bruker SMART CCD area-detector diffractometer
Absorption correction: none
10040 measured reflections
1873 independent reflections
1591 reflections with *I* > 2σ(*I*)
*R*_int_ = 0.031

#### Refinement

*R*[*F* ^2^ > 2σ(*F* ^2^)] = 0.030
*wR*(*F* ^2^) = 0.094
*S* = 1.08
1873 reflections
100 parameters
H-atom parameters constrained
Δρ_max_ = 0.32 e Å^−3^
Δρ_min_ = −0.25 e Å^−3^

### 

Data collection: *SMART-NT* (Bruker, 1998 ▶); cell refinement: *SAINT-Plus* (Bruker, 1999 ▶); data reduction: *SAINT-Plus*; program(s) used to solve structure: *SHELXS97* (Sheldrick, 2008 ▶); program(s) used to refine structure: *SHELXL97* (Sheldrick, 2008 ▶); molecular graphics: *ORTEP-3* (Farrugia, 1997 ▶) and *Mercury* (Macrae *et al.*, 2006 ▶); software used to prepare material for publication: *WinGX* (Farrugia, 1999 ▶).

## Supplementary Material

Crystal structure: contains datablocks I, global. DOI: 10.1107/S160053680800665X/tk2250sup1.cif

Structure factors: contains datablocks I. DOI: 10.1107/S160053680800665X/tk2250Isup2.hkl

Additional supplementary materials:  crystallographic information; 3D view; checkCIF report

## Figures and Tables

**Fig. 1.**
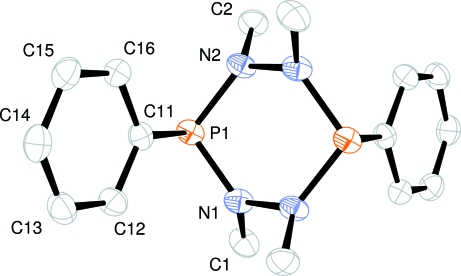
Molecular structure of (I) drawn with displacement ellipsoids at the 50% probability level. Hydrogen atoms have been omitted for clarity.

## supplementary crystallographic information

### Comment

Compound (I) has a centre of symmetry and the six-membered ring adopts a chair conformation with the phenyl groups on the phosphorous atoms being *trans* to each other. Reddy *et al.* (1994) have observed in related compounds, *e.g.* [HNP(Et)N(Me)]_2_ and [HNP(Ph)N(Me)]_2_, that the chair conformation was favoured over the boat conformation and was seen to readily crystallize. Bond lengths and angles in (I), with values for N—N 1.4321 (17), P—N 1.6953 (14) and 1.6984 (12), and P—C_arom_ 1.8412 (15) Å, are in the ranges observed in related structures (Reddy *et al.*, 1994, 1995).

### Experimental

In an attempt to crystallize bis(diphenylphosphino)dimethylhydrazine (II) (for synthesis see Reddy *et al.*, 1995), the worked-up diethylether reaction mixture was concentrated and kept at -20 °C for two days. Two small crystals were formed and on analysis of one of the crystals, (I) was identified. Analysis of the ^31^P NMR spectrum of (II) showed (I) to be present in less than 5%. Further analysis of (I) was not attempted due to the small amount of material available.

### Refinement

The H atoms were positioned geometrically and allowed to ride on their respective parent atoms, with C—H = 0.93 (Ar—H) or 0.96 (CH_3_) Å, and with *U*_eq_ = 1.2 (Ar—H) or 1.5 (CH_3_) *U*_eq_(C).

### Crystal data

**Table:**

C_16_H_22_N_4_P_2_	*F*_000_ = 704
*M**_r_* = 332.32	*D*_x_ = 1.286 Mg m^−^^3^
Orthorhombic, *P**b**c**a*	Mo *K*α radiation λ = 0.71073 Å
Hall symbol: -P 2ac 2ab	Cell parameters from 899 reflections
*a* = 13.2879 (16) Å	θ = 3.0–28.2º
*b* = 7.5426 (9) Å	µ = 0.26 mm^−^^1^
*c* = 17.125 (2) Å	*T* = 173 (2) K
*V* = 1716.4 (4) Å^3^	Plates, colourless
*Z* = 4	0.38 × 0.27 × 0.26 mm

### Data collection

**Table:**

Bruker SMART CCD area-detector diffractometer	1591 reflections with *I* > 2σ(*I*)
Radiation source: fine-focus sealed tube	*R*_int_ = 0.031
Monochromator: graphite	θ_max_ = 27.0º
*T* = 173(2) K	θ_min_ = 2.4º
φ and ω scans	*h* = −15→16
Absorption correction: none	*k* = −7→9
10040 measured reflections	*l* = −21→21
1873 independent reflections	

### Refinement

**Table:**

Refinement on *F*^2^	Secondary atom site location: difference Fourier map
Least-squares matrix: full	Hydrogen site location: inferred from neighbouring sites
*R*[*F*^2^ > 2σ(*F*^2^)] = 0.030	H-atom parameters constrained
*wR*(*F*^2^) = 0.094	*w* = 1/[σ^2^(*F*_o_^2^) + (0.0506*P*)^2^ + 0.5379*P*] where *P* = (*F*_o_^2^ + 2*F*_c_^2^)/3
*S* = 1.08	(Δ/σ)_max_ = 0.005
1873 reflections	Δρ_max_ = 0.32 e Å^−^^3^
100 parameters	Δρ_min_ = −0.25 e Å^−^^3^
Primary atom site location: structure-invariant direct methods	Extinction correction: none

### Special details

**Table:**

Geometry. All e.s.d.'s (except the e.s.d. in the dihedral angle between two l.s. planes) are estimated using the full covariance matrix. The cell e.s.d.'s are taken into account individually in the estimation of e.s.d.'s in distances, angles and torsion angles; correlations between e.s.d.'s in cell parameters are only used when they are defined by crystal symmetry. An approximate (isotropic) treatment of cell e.s.d.'s is used for estimating e.s.d.'s involving l.s. planes.
Refinement. Refinement of *F*^2^ against ALL reflections. The weighted *R*-factor *wR* and goodness of fit *S* are based on *F*^2^, conventional *R*-factors *R* are based on *F*, with *F* set to zero for negative *F*^2^. The threshold expression of *F*^2^ > σ(*F*^2^) is used only for calculating *R*-factors(gt) *etc*. and is not relevant to the choice of reflections for refinement. *R*-factors based on *F*^2^ are statistically about twice as large as those based on *F*, and *R*- factors based on ALL data will be even larger.

### Fractional atomic coordinates and isotropic or equivalent isotropic displacement parameters (Å^2^)

**Table:**

	*x*	*y*	*z*	*U*_iso_*/*U*_eq_	
C1	−0.08579 (13)	0.3217 (2)	−0.05297 (9)	0.0359 (4)	
H1A	−0.0364	0.4122	−0.0624	0.054*	
H1B	−0.1442	0.3737	−0.0292	0.054*	
H1C	−0.1044	0.2675	−0.1016	0.054*	
C2	0.17104 (11)	−0.0731 (2)	−0.08775 (8)	0.0336 (3)	
H2A	0.2138	0.0277	−0.0961	0.050*	
H2B	0.1272	−0.0878	−0.1319	0.050*	
H2C	0.2116	−0.1775	−0.0815	0.050*	
C11	0.09375 (10)	0.14618 (18)	0.11718 (8)	0.0257 (3)	
C12	0.03662 (11)	0.25669 (19)	0.16564 (8)	0.0295 (3)	
H12	−0.0127	0.3288	0.1438	0.035*	
C13	0.05251 (13)	0.2604 (2)	0.24603 (9)	0.0339 (3)	
H13	0.0135	0.3338	0.2775	0.041*	
C14	0.12622 (12)	0.1549 (2)	0.27919 (9)	0.0362 (4)	
H14	0.1359	0.1554	0.3330	0.043*	
C15	0.18563 (12)	0.0482 (2)	0.23183 (9)	0.0372 (4)	
H15	0.2359	−0.0215	0.2539	0.045*	
C16	0.17020 (11)	0.0452 (2)	0.15125 (9)	0.0307 (3)	
H16	0.2112	−0.0248	0.1199	0.037*	
N1	−0.04376 (10)	0.18785 (15)	−0.00091 (7)	0.0277 (3)	
N2	0.11088 (9)	−0.04525 (16)	−0.01743 (7)	0.0277 (3)	
P1	0.08233 (3)	0.16498 (5)	0.01035 (2)	0.02618 (14)	

### Atomic displacement parameters (Å^2^)

**Table:**

	*U*^11^	*U*^22^	*U*^33^	*U*^12^	*U*^13^	*U*^23^
C1	0.0509 (10)	0.0263 (8)	0.0304 (8)	0.0060 (7)	−0.0051 (7)	0.0052 (6)
C2	0.0319 (7)	0.0386 (8)	0.0302 (7)	0.0019 (6)	0.0104 (6)	−0.0022 (6)
C11	0.0285 (7)	0.0237 (7)	0.0250 (7)	−0.0039 (5)	0.0019 (5)	0.0006 (5)
C12	0.0330 (7)	0.0262 (7)	0.0292 (7)	0.0013 (6)	0.0007 (6)	0.0004 (6)
C13	0.0375 (8)	0.0345 (8)	0.0298 (8)	−0.0014 (6)	0.0017 (6)	−0.0076 (7)
C14	0.0391 (9)	0.0413 (9)	0.0283 (7)	−0.0057 (7)	−0.0076 (6)	−0.0036 (6)
C15	0.0339 (8)	0.0385 (9)	0.0392 (8)	0.0016 (7)	−0.0129 (6)	−0.0017 (7)
C16	0.0269 (7)	0.0306 (7)	0.0346 (7)	−0.0002 (6)	−0.0009 (6)	−0.0042 (6)
N1	0.0323 (6)	0.0226 (6)	0.0282 (6)	0.0037 (5)	0.0036 (5)	0.0076 (5)
N2	0.0314 (6)	0.0252 (6)	0.0265 (6)	0.0025 (5)	0.0099 (5)	0.0015 (5)
P1	0.0307 (2)	0.0230 (2)	0.0248 (2)	0.00033 (14)	0.00652 (14)	0.00337 (13)

### Geometric parameters (Å, °)

**Table:**

C1—N1	1.4583 (18)	C12—H12	0.9300
C1—H1A	0.9600	C13—C14	1.384 (2)
C1—H1B	0.9600	C13—H13	0.9300
C1—H1C	0.9600	C14—C15	1.389 (2)
C2—N2	1.4605 (17)	C14—H14	0.9300
C2—H2A	0.9600	C15—C16	1.395 (2)
C2—H2B	0.9600	C15—H15	0.9300
C2—H2C	0.9600	C16—H16	0.9300
C11—C16	1.398 (2)	N1—N2^i^	1.4321 (17)
C11—C12	1.400 (2)	N1—P1	1.6953 (14)
C11—P1	1.8412 (15)	N2—N1^i^	1.4321 (17)
C12—C13	1.393 (2)	N2—P1	1.6984 (12)
			
N1—C1—H1A	109.5	C12—C13—H13	120.0
N1—C1—H1B	109.5	C13—C14—C15	119.73 (14)
H1A—C1—H1B	109.5	C13—C14—H14	120.1
N1—C1—H1C	109.5	C15—C14—H14	120.1
H1A—C1—H1C	109.5	C14—C15—C16	120.24 (14)
H1B—C1—H1C	109.5	C14—C15—H15	119.9
N2—C2—H2A	109.5	C16—C15—H15	119.9
N2—C2—H2B	109.5	C15—C16—C11	120.72 (14)
H2A—C2—H2B	109.5	C15—C16—H16	119.6
N2—C2—H2C	109.5	C11—C16—H16	119.6
H2A—C2—H2C	109.5	N2^i^—N1—C1	114.56 (12)
H2B—C2—H2C	109.5	N2^i^—N1—P1	120.94 (9)
C16—C11—C12	118.12 (13)	C1—N1—P1	121.23 (10)
C16—C11—P1	121.12 (11)	N1^i^—N2—C2	114.43 (11)
C12—C11—P1	119.90 (11)	N1^i^—N2—P1	120.04 (9)
C13—C12—C11	121.04 (14)	C2—N2—P1	119.18 (10)
C13—C12—H12	119.5	N1—P1—N2	106.49 (6)
C11—C12—H12	119.5	N1—P1—C11	101.67 (6)
C14—C13—C12	120.08 (14)	N2—P1—C11	100.84 (6)
C14—C13—H13	120.0		
			
C16—C11—C12—C13	−2.7 (2)	N2^i^—N1—P1—C11	−66.99 (11)
P1—C11—C12—C13	−172.18 (11)	C1—N1—P1—C11	134.55 (11)
C11—C12—C13—C14	0.5 (2)	N1^i^—N2—P1—N1	−37.75 (13)
C12—C13—C14—C15	1.3 (2)	C2—N2—P1—N1	112.73 (11)
C13—C14—C15—C16	−1.0 (2)	N1^i^—N2—P1—C11	68.00 (11)
C14—C15—C16—C11	−1.3 (2)	C2—N2—P1—C11	−141.52 (11)
C12—C11—C16—C15	3.1 (2)	C16—C11—P1—N1	148.76 (12)
P1—C11—C16—C15	172.42 (12)	C12—C11—P1—N1	−42.10 (12)
N2^i^—N1—P1—N2	38.17 (13)	C16—C11—P1—N2	39.20 (13)
C1—N1—P1—N2	−120.30 (12)	C12—C11—P1—N2	−151.65 (11)

Symmetry codes: (i) −*x*, −*y*, −*z*.
